# Leisure sedentary behaviour increases the risk of venous thromboembolism: a Mendelian randomisation study

**DOI:** 10.1186/s12872-023-03395-5

**Published:** 2023-07-18

**Authors:** Liang Chen, Guochang You, Zhenmei Yang, Runnan Shen, Rong Zhang, Dongxi Zhu, Linlu Wang, Shen Lin, Lin Lv, Kai Huang

**Affiliations:** 1 Department of Critical Care Medicine, Sun Yat-Sen Memorial Hospital, Sun Yat-Sen University, Guangzhou, Guangdong Province P. R. China; 2 Zhongshan School of Medicine, Sun Yat-Sen University, Guangzhou, Guangdong Province P. R. China; 3 Department of Breast Oncology, Sun Yat-Sen Memorial Hospital, Sun Yat-Sen University, Guangzhou, Guangdong Province P. R. China; 4 Department of Anesthesiology, Sun Yat-Sen Memorial Hospital, Sun Yat-Sen University, Guangzhou, Guangdong Province P. R. China; 5 Department of Cardiovascular Surgery, Sun Yat-Sen Memorial Hospital, Sun Yat-Sen University, No.33, Yingfeng Road, Haizhu District, Guangzhou, Guangdong Province P. R. China

**Keywords:** Mendelian randomisation analysis, Venous thromboembolism, Leisure sedentary behaviours, Causality

## Abstract

**Background:**

Venous thromboembolism (VTE) is a substantial contributor to the global burden of disease. Observational studies have suggested that leisure sedentary behaviours (LSB) are related to the risk of VTE; however, the causal role of LSB in VTE remains unclear.

**Methods:**

Using data obtained from genome-wide association studies in the UK Biobank (*N* = 422,218), we identified 84, 21, and 4 single nucleotide polymorphisms (SNPs) related to sedentary television (TV) watching, computer use, and driving, respectively. These SNPs were employed as instrumental variables. Summary statistics for SNP-VTE associations was obtained from the FinnGen study (5,403 cases and 130,235 controls). Two-sample Mendelian randomisation (MR) analyses were performed using inverse-variance weighted (IVW), MR-Egger,weighted median, and weighted mode approaches. Sensitivity analyses were conducted to ensure robustness of the results.

**Results:**

The main IVW approach demonstrated a positive association between the genetically predicted sedentary TV watching and the risk of VTE [odds ratio (OR):1.35, 95% confidence interval (CI):1.02—1.80, *P* = 0.039]. However, no significant association was observed for genetically predicted sedentary computer use or driving and VTE risk. The results from our series of sensitivity analyses, including Cochran’s Q test, MR-Egger intercept test, and MR-Pleiotropy RESidual Sum and Outlier method, further supported these findings.

**Conclusion:**

This study provides evidence of an association between genetically predicted sedentary TV watching and the risk of VTE. Further studies are required to elucidate the underlying causal mechanisms.

**Supplementary Information:**

The online version contains supplementary material available at 10.1186/s12872-023-03395-5.

## Background

Venous thromboembolism (VTE), comprising deep vein thrombosis (DVT) and pulmonary embolism (PE), is globally the third most frequent acute cardiovascular syndrome after myocardial infarction and stroke. It is also an important factor contributing to the global burden of cardiovascular disease [1–3]. VTE affects approximately 10 million people worldwide every year [4, 5]. PE, causing up to 300,000 deaths per year in the United States, is among the main causes of cardiovascular-related deaths [4]. In addition to recurrent VTE and bleeding caused by anticoagulant use, clinical sequelae after VTE include post-thrombotic and post-pulmonary embolism syndromes [6–8]. These clinical conditions greatly reduce the quality of life and result in a serious economic healthcare burden; their estimated burden on the European Union-28’s healthcare systems is EUR 8.5 billion [9, 10]. Considering the high prevalence and disease burden of VTE, it is necessary to investigate the potential modifiable risk factors for this disease and tailor feasible intervention strategies for primary and secondary preventions.

Leisure sedentary behaviours (LSB) refer to any waking activity encompassing an energy expenditure of less than 1.5 metabolic equivalents of task (METs) in a seated, reclined, or lying posture, such as sedentary television (TV) watching, computer use, and driving [11]. LSB are among the leading modifiable risk factors for cardiovascular disease and related mortality worldwide [12–14], and prolonged sedentary time is associated with common cardio-metabolic and inflammatory biomarkers [15]. Furthermore, the association between LSB and VTE risk has been increasingly reported. Meta-analyses of observational prospective cohort studies have demonstrated an association between prolonged TV watching and increased risk of VTE [16, 17], and similar associations have been observed for other LSB, such as computer use and driving [18–20]. However, the epidemiological conclusions remain contradictory. For instance, a prospective study with a large biracial US cohort reported no association between TV watching and the risk of VTE, independently of physical activity [21]. Additionally, conventional epidemiological studies show several limitations, including deficient statistical robustness owing to small sample size, confusing results from biases or reverse causality, and varying result interpretations. Consequently, whether LSB is associated with VTE risk remains unclear.

Mendelian randomisation (MR), which uses genetic variants and follows the law of independent assortment, can assess the causality of an observed association between a modifiable exposure or risk factor and a clinically relevant outcome [22, 23]. MR analysis minimises confusion and avoids deviation caused by reverse causality. In MR, the evaluation of genotypes determines predisposition, precedes the onset of diseases, and is free from the impact of lifestyle and environment factors [24]. As an extension of the MR method, the two-sample MR analysis allows the use of summary-level statistics of genome-wide association studies (GWASs) without requiring direct analysis of individual-level data. Based on available data from GWASs in the UK Biobank [25], the genetic variants associated with LSB can be set as instrumental variables (IVs) to explore the impact of LSB on VTE, excluding confounders such as physical activity and smoking status from the analysis. In this study, we performed a two-sample MR study to elucidate the potential impact of LSB (sedentary TV watching, computer use, and driving) on VTE.

## Methods

### Data sources

Data on the association between single nucleotide polymorphisms (SNPs) and LSB were acquired from large-scale GWAS meta-analyses in the UK Biobank. This cohort was composed of 422,218 community-dwelling adults of European ancestry. The three phenotypes of LSB considered in this study were TV watching, computer use (except that at work), and driving. The sedentary time spent in each LSB had been ascertained in an interview. At the time of the first assessment (interview), 45.7% of the participants were male, and the average age was 57.4 (SD 8.0) years. Mean reported daily leisure time spent on each LSB was 2.8 h (SD 1.5) for TV watching, 1.0 h (SD 1.2) for computer use, and 0.9 h (SD 1.0) for driving. Further details of this study have been previously described [25]. LSB associated with genetic variants were adjusted for age, sex, body mass index, smoking status, hypertension, diabetes, Townsend deprivation index, physical activity levels, alcohol use per week, and years of education.

 Summary statistics for SNP-VTE associations were retrieved from the FinnGen study, which comprised 5,403 VTE patients and 130,235 control cases. Details on the participating biobank/cohorts, genotypes, and methods of data collection and analysis are available on the FinnGen website (https://finngen.gitbook.io/documentation/).

### Ethical approval

This study was conducted using publicly available de-identified data from participants in studies with appropriate approvals from ethical committees regarding human experimentation. No additional ethical approval was required for this study.

### Selection of genetic instruments

The MR approach was based on a few core assumptions regarding the genetic variants used as IVs: they are associated with LSB exposure [26], are independent of the potential confounders of the association between LSB exposure and VTE outcome, and only affect VTE outcome through LSB exposure. Therefore, SNPs were considered IVs only if they satisfied these three core assumptions as independent genetic predictors. Rigorous filtering procedures were conducted to ensure SNP quality prior to MR analysis [27]. More specifically, we used summary estimations of 152, 37, and 4 significant SNPs (*P* < 1 × 10^–8^) for TV watching, computer use, and driving, respectively, that were identified using the original GWASs. Selected SNPs were grouped with a 10,000 kb window, and SNPs with larger *P* values at a threshold of linkage disequilibrium (LD) R^2^ over 0.001 were excluded from the analysis. Additionally, 50 SNPs were excluded from TV watching and 13 from computer use using the LD analysis. SNPs which were significantly associated with VTE outcome (at the genome-wide significance threshold *P* < 5 × 10^–8^) were then excluded. For any specifically required SNP missing from the VTE datasets, we used proxies with strong LD (R^2^ > 0.8) to replace them. The strength of the genetic instruments used as IVs was further evaluated using F-statistics, where SNPs with an F < 10 were excluded. Additionally, ambiguous and palindromic SNPs (EAF > 0.42) were excluded with harmonizing processes. Detailed information relative to excluded SNPs is shown in Supplementary Table 1. The final IV dataset was composed of 84, 21, and 4 SNPs for sedentary TV watching, computer use, and driving, respectively.

### Mendelian randomisation analyses

Two-sample MR analysis was performed using the inverse-variance weighted (IVW) method with multiplicative random effects, set as the main analysis for causal effect estimation. This approach assumes non-associated and independent effects of multiple genetic variants [28]. The estimated results of genetically predicted LSB exposure on VTE outcome were calculated using the Wald ratio method. Furthermore, complementary sensitivity analyses were conducted to verify the validity and robustness of the calculated effects. More explicitly, the weighted median approach was applied, which provided a robust and consistent effect estimation if at least half of the information in the analysis comes from SNPs that are valid instrumental variables [29]. MR-Egger analysis was also conducted to provide an effect estimate where whole genetic variants exhibit pleiotropic effects not related to the variant-exposure association [30]. Nevertheless, the IVW and MR-Egger analyses commonly show low precision (wider confidence intervals). The weighted mode method was thus implemented; it showed the most intuitive validity conditions that are important for triangulating point estimates. Power calculations were also conducted (http://cnsgenomics.com/shiny/mRnd/) [31]. The calculation was post hoc, and the related parameters in the website were set as follows (outcome type: binary outcome, sample size: 135,638 [5,403 + 130,235]), type-I error rate: 0.05, proportion of cases in the study: 0.0398 [5,403/135,638], true odds ratio of the outcome variable per standard deviation of the exposure variable: 1.35 [result of IVW method for sedentary TV watching], proportion of variance explained for the association between the SNP and the exposure variable: 0.0105).

Effect heterogeneity was evaluated using the Cochran’s Q test, and *P* < 0.05 was defined as significantly heterogeneous. To detect the pleiotropic effects of the IVs, MR-Egger was used with the Egger intercept, where a significant deviation of from zero suggests a horizontal pleiotropy. Additionally, directional pleiotropy was detected through asymmetry and precision in funnel plots of the MR estimate. Leave-one-out analysis was conducted to evaluate the sensitivity of each genetic variant and to determine whether the estimated results of the IVW approach were biased by any specific SNP. The risk estimates of each SNP for the LSB outcome were calculated and presented as forest plots. Furthermore, the MR-Pleiotropy RESidual Sum and Outlier (MR-PRESSO) method was applied to eliminate SNPs with potential pleiotropy (horizontal pleiotropy < 50%).

To exclude the biases caused by potential risk confounders, we manually scanned potential secondary phenotypes related to our selected SNPs using PhenoScanner (www.phenoscanner.medschl.cam.ac.uk), a platform with extensive data on associations between genotype and phenotype [32]. SNPs with any association (*P* < 1 × 10^–5^) with these potential confounding factors at genome-wide significance were excluded. The potential risk factors considered included obesity, high-density lipoprotein cholesterol (HDL), triglycerides, smoking, and cardiovascular disease.

Because these sedentary behaviours are social exposures, the likelihood of a violation of the independence assumption becomes greater owing to confounding factors [33]; thus, conducting subsequent sensitivity analyses is important. We used hair colour as negative control for population structure, which is publicly available for the UK Biobank through OpenGWAS (https://gwas.mrcieu.ac.uk/datasets/ukb-d-1747_5/) [34, 35]. Furthermore, the within-family consortia GWAS of physical activity (https://gwas.mrcieu.ac.uk/datasets/ieu-b-4859/) was used as a control exposure to further validate our findings. Owing to the small sample size, a more indicative *p*-value (5 × 10^–6^) was chosen to select IVs. Subsequently, after the same population assumption test of FinnGen and UK Biobank cohorts was performed using MRSamePopTest [36], a meta-analysis of the two populations was conducted as another sensitivity analysis.

### Statistics

All analyses were conducted using the Two-Sample MR package (version 0.4.25) and MR-PRESSO (version 1.0) in the R software (version 3.6.1). Statistical significance was defined as a two-sided *p*-value ≤ 0.05.

## Results

We identified 145, 36, and 4 SNPs as IVs for leisure TV watching, computer use, and driving, respectively, using data from GWASs in the UK Biobank study. After grouping, 95, 23, and 4 SNPs, respectively, remained for further screening. Following overall screening, 84, 21, and 4 SNPs were obtained as IVs for sedentary TV watching, sedentary computer use, and sedentary driving, respectively. Supplementary Table 2 presents summary information of our chosen SNPs for the three LSB in this MR study. For SNPs that failed to overlap between the LSB and VTE datasets, proxy SNPs were used. No outlier SNPs were detected with MR-PRESSO analyses (*P* > 0.05, Supplementary Table 3). All included SNPs showed F-statistics > 10, indicating weak instrument bias (Supplementary Table 4).

As shown in Table 1, genetically predicted leisure TV watching was positively associated with VTE risk [odds ratio (OR):1.35, 95% confidence interval (CI):1.02—1.80, *P* = 0.039, calculated using IVW]. Furthermore, the MR-Egger intercept (-0.012, Table 2) provided weak evidence of horizontal pleiotropy in the analyses (*P*_intercept_ = 0.287), and non-significant heterogeneity in the IVW estimates from the results of the Cochran’s Q test (*P* = 0.089). Additionally, no outlier SNPs were identified in the scatter plot, leave-one-out, and funnel analyses for the three LSB phenotypes (Supplementary Figs. 1-9). The scatter plots of the associations between SNPs-LSB and SNPs-VTE showed that the heterogeneity of genetic IVs was balanced at near zero, and no violation was caused by horizontal pleiotropy (MR-Egger intercept passed through zero). The leave-one-out analysis indicated that the three sedentary phenotypes of LSB and the VTE risk remained consistent after exclusion of one SNP at a time, which identified no outlying variants. The MR regression funnel plots were symmetrical, implying a minimal deviation from directional pleiotropy.

**Table 1:** MR estimates of the causal relationship between leisure sedentary behaviors and the risk of VTE

Exposure	Methods	VTE	
		OR (95% CI)	*P value*
Television watching	IVW	1.35 (1.02, 1.80)	0.039
	WM	1.48 (0.99, 2.23)	0.056
	MR-Egger	2.85 (0.71, 11.42)	0.144
	Weighted mode	1.95 (0.71, 5.33)	0.196
Computer use	IVW	0.97 (0.57, 1.65)	0.919
	WM	1.28 (0.61, 2.65)	0.513
	MR-Egger	0.21 (0.003, 14.46)	0.481
	Weighted mode	1.41 (0.42, 4.79)	0.584
Driving	IVW	1.21 (0.16, 9.08)	0.855
	WM	2.08 (0.44, 9.95)	0.357
	MR-Egger	2376.18 (0.0001, 3.18 × 10^10^)	0.451
	Weighted mode	3.38 (0.52, 21.84)	0.291

*MR* Mendelian randomisation, *VTE* Venous thromboembolism, *IVW* Random-effect inverse variance weighted, *WM* Weighted median, *OR* Odds ratio, *CI* Confidence interval

**Table 2:** Sensitivity analysis of the associations between LSB and the risk of VTE

Outcome	Exposure	Cochran's Q test		MR-Egger	
		Q value	*P value*	Intercept	*P value*
VTE	Television watching	100.801	0.089	-0.012	0.287
	Computer use	14.227	0.819	0.024	0.485
	Driving	7.940	0.047	-0.117	0.457

*LSB* Leisure sedentary behaviors, *MR* Mendelian randomisation, *VTE* Venous thromboembolism

Nevertheless, little evidence supported the causal effect of genetically predicted sedentary computer use and driving on the risk of VTE. Comprehensive sensitivity analyses showed little heterogeneity or pleiotropy-biased MR estimates. Heterogeneity was observed with Cochran’s Q test (*P* = 0.047) in the sensitivity analysis of leisure driving, which was acceptable as we used the random effects IVW as the main approach [37]. Forest plots for leisure TV watching, computer use, and driving are shown in Supplementary Figs. 10-12. Furthermore, through manual scanning of each selected SNP to determine whether the potential risk factors violated the significance estimation in PhenoScanner, rs13107325 and rs749671 were identified. After excluding these SNPs, the estimates remained consistent with previous results (OR: 1.34, 95% CI: 1.00—1.79, *P* = 0.048), indicating that the causality between LSB and the risk of VTE was not biased by potential risk confounders. However, we had limited power (73%) to test causal effects of these behaviours on VTE risk. Two additional sensitivity analyses, including black hair colour as negative control and physical activity as control exposure, further supported our findings (Supplementary Table 5). Although there was no significant result from the physical activity group, the beta value was generally in the opposite direction to that of sedentary behaviours. Additionally, the MRSamePopTest of the FinnGen and UK Biobank cohorts showed no difference in the LSB effects between the two populations (all *P* > 0.05) (Supplementary Table 6). The risk trend of the meta-analysis was consistent with our findings, although without statistical significance (Supplementary Fig. 13).

## Discussion

To the best of our knowledge, this is the first large-scale MR analysis to assess the causal relationship between sedentary TV watching, computer use, and driving and the risk for VTE using extensive genetic data from available databases. As demonstrated through our results, genetically predicted sedentary TV watching is positively associated with the risk of VTE. However, no evidence was found to support causality between sedentary computer use or sedentary driving and VTE.

The findings of our study are in agreement with those from several observational studies. Prospective research with the Atherosclerosis Risk in Communities (ARIC) cohort reported that a higher frequency of watching TV during leisure time was associated with increased VTE risk independently of physical activity and obesity [38]. The determination of TV watching frequency in the ARIC cohort was qualitative ("never or seldom", "sometimes", "often, or "very often"), not defining the duration. However, qualitative frequency can be positively associated with the amount of time spent watching TV from a practical perspective. The JACC (Japan Collaborative Cohort) study showed that time spent watching TV is associated with the risk of PE mortality, with multivariable hazard ratios of 1.7 (95% CI: 0.9—3.0) for those watching TV for 2.5 to 4.9 h/d, and 2.5 (95% CI: 1.2—5.3) for those ≥ 5 h/d, compared with individuals watching TV for < 2.5 h/d [39]. Conversely, a recent study with a large US cohort showed that TV watching is not associated with VTE risk, with no difference in association when adjusting for physical activity levels [21]. Nevertheless, several limitations of these observational studies should be considered. Although in these prospective cohort studies several potential confounding factors were adjusted to reduce bias, residual confounders were difficult to adjust and had different impacts on the results, such as air pollution, diet, and genetic risk factors, that could drive sedentary behaviours or induce VTE events [40, 41]. Furthermore, rough estimates of sedentary TV watching time were mainly conducted through questionnaires (mostly self-reported assessments), which might have skewed the results with reduced accuracy [42]. Additionally, the main population, inclusion criteria, and loss of follow-up in these studies were distinct. Furthermore, inclusion in the study primarily relied on voluntary participation, which may have resulted in selection bias [43, 44].

Other studies support our conclusion, suggesting that prolonged TV watching could promote venous stasis, elevating the tendency of thrombosis by influencing the levels of circulating haemostatic and inflammatory factors. However, the underlying mechanisms of these observations remain poorly understood. In a longitudinal cohort study conducted by Hamer et al., there was an association between TV watching (a major part of sedentary behaviour) and increased white blood cell counts and fibrinogen concentration, after adjustment for covariates. Additionally, at follow up, increased TV watching was related to increased C-reactive protein levels and white blood cell count [45]. Considering that high fibrinogen concentration is an important risk factor for thrombotic diseases, and that inflammation is implicated in VTE, the elevation of these acute-phase reactants and coagulation markers could be related to an increased risk of vascular diseases, particularly VTE [45–47]. Furthermore, several studies have suggested cross-sectional associations between TV watching or objective sedentary time and markers of low-grade inflammation [48, 49]. A randomised controlled trial conducted by Howard et al. suggested that uninterrupted sitting during sedentary time increases fibrinogen levels and reduces plasma volume, with concomitant increases in haemoglobin levels and haematocrit, which have procoagulant effects and contribute to increased risk of thrombosis [50]. Genetically predicted endogenous haemoglobin is a key red blood cell trait causing VTE with a detrimental effect on the general population. Therefore, increased haemoglobin levels promoted by prolonged sedentary sitting could increase the risk of thromboembolic events [51]. In addition, TV watching occurs primarily in the evening after the main meal. This might lead to repetitive negative effects of postprandial blood sugar and lipid levels on cardiovascular structures, increasing the metabolic risk for VTE, especially if the duration of TV watching is increased by prolonged sitting [52–54]. Moreover, evidence suggests that the vascular and metabolic consequences of physical activity are mainly regulated through peripheral tissues and cells, including muscle, adipose tissues, and endothelial and inflammatory cells [13, 55]. Physical inactivity (mainly sedentary TV watching) may cause VTE through a complex network of reactions. More research on animal or human models is required to determine the pathophysiological changes caused by sedentary TV watching, which could lead to new preventive and therapeutic options.

The main finding of our study is that genetically predicted sedentary TV watching is associated with an increased risk of VTE. Moreover, we found little evidence for a causal relationship between sedentary computer use or driving and the risk of VTE. The large difference in the sedentary time spent on each of these three behaviours in the selected population could partly explain these differences. The average sedentary TV watching time is almost three times higher than that of sedentary computer use or driving (mean daily sedentary TV watching time was 2.8 h [SD 1.5], whereas that of leisure computer use was 1.0 h [SD 1.2] and that of driving time was 0.9 h [SD 1.0]). A dose–response meta-analysis comprising 1,331,468 participants showed an association between the risk of all-cause mortality, cardiovascular disease-associated mortality, and incidence of type 2 diabetes with higher levels of sedentary behaviour time (mainly TV watching time), independently of physical activity, and the associations appeared to be non-linear with a change in gradient [56]. Considering that there might be a dose–response relationship between exposure time and the outcome, as well as that exposure duration in our study may not have exceeded the potential hazard threshold, we cannot exclude the relationship between these two exposures (sedentary computer use or driving) and VTE. Furthermore, our study currently only provided guidance to "sit less" and "break prolonged sitting times" during TV watching behaviour in leisure time, and it is difficult to provide a quantitative recommendation. Considering the severe socio-economic and healthcare burden of VTE, it is crucial to implement appropriate primary preventive measures and promote a healthy lifestyle throughout life [57]. Based on the findings of our study, sedentary TV watching is one modifiable risk factor for VTE; therefore, the recommendations to "sit less" and "break prolonged sitting times" in this sedentary behaviour are consistent with other guidelines [58].

This study has several limitations. First, our analysis was restricted to individuals of European descent, and whether these findings are applicable to other ethnic groups requires further determination. Second, our analysis relied on public data, which cannot be used to further stratified analyses (comprising the DVT and PE subgroups). Third, because of the results without statistical significance in our study, we could not completely exclude an association between these sedentary behaviours (computer use and driving) and VTE; this may result from the insufficient statistical power caused by the relatively small sample size (< 80%). Finally, considering that the biological mechanisms of several of the selected IVs are still unclear, directly assessing the horizontal pleiotropy that might bias the MR results is impractical. Nonetheless, our sensitivity analysis showed no evidence of pleiotropy. Additionally, with the purpose of excluding the biases caused by potential risk confounders on the causal estimations, we excluded SNPs associated with any of the potential risk factors, including obesity, HDL, triglycerides, smoking, cardiovascular diseases, and LSB associated with genetic variants for IVs. We also adjusted for age, sex, body mass index, smoking status, hypertension, diabetes, Townsend deprivation index, physical activity levels, alcohol use per week, and years of education, which ensured that the MR approach in our study minimised the biases of these confounding factors on the causality effects evaluated.

## Conclusions

The present MR analysis demonstrated a causal relationship between genetically predicted sedentary TV watching and VTE. Further studies are required to test and verify our findings and clarify the underlying mechanisms.

## Supplementary Information

**Additional file 1: Supplementary Figure 1.** Scatter plot from genetically predicted sedentary TV watching on risk of VTE. **Supplementary Figure 2.** Scatter plot from genetically predicted sedentary computer use on risk of VTE. **Supplementary Figure 3.** Scatter plot from genetically predicted sedentary driving on risk of VTE. **Supplementary Figure 4.** Leave-one-out plot from genetically predicted sedentary TV watching on risk of VTE. **Supplementary Figure 5.** Leave-one-out plot from genetically predicted sedentary computer use on risk of VTE. **Supplementary Figure 6.** Leave-one-out plot from genetically predicted sedentary driving on risk of VTE. **Supplementary Figure 7.** Funnel plot from genetically predicted sedentary TV watching on risk of VTE. **Supplementary Figure 8.** Funnel plot from genetically predicted sedentary computer use on risk of VTE. **Supplementary Figure 9.** Funnel plot from genetically predicted sedentary driving on risk of VTE. **Supplementary Figure 10.** Forest plot from genetically predicted sedentary TV watching on risk of VTE. **Supplementary Figure 11.** Forest plot from genetically predicted sedentary computer use on risk of VTE. **Supplementary Figure 12.** Forest plot from genetically predicted sedentary driving on risk of VTE. **Supplementary Figure 13.** Forest plot of the meta-analysis for genetically predicted sedentary TV watching on risk of VTE in FinnGen and UK Biobank populations. **Supplementary Table 1.** Summary information on the excluded SNPs for LSB in the present MR study. **Supplementary Table 2.** Summary information on the SNPs used as genetic instruments for LSB in the present MR study. **Supplementary Table 3.** MR-PRESSO results. **Supplementary Table 4.** F-statistics of LSB and physical activity. **Supplementary Table 5.** MR estimates of the causal relationship between black hair color, physical activity and the risk of VTE. **Supplementary Table 6.** MRSamePopTest in the FinnGen and UK Biobank population.

## Data Availability

The datasets generated and/or analyzed during the current study are publicly available in the following GWAS repository (https://doi.org/10.17632/mxjj6czsrd.1, https://www.finngen.fi/en/access_results).

## Abbreviations

ARIC: Atherosclerosis Risk in Communities
CI: Confidence interval
DVT: Deep vein thrombosis
EUR: European Monetary Unit
GWASs: Genome-wide association studies
HDL: High-density lipoprotein cholesterol
IVs: Instrumental variables
IVW: Inverse-variance weighted
LD: Linkage disequilibrium
LSB: Leisure sedentary behaviors
METs: Metabolic equivalents of task
MR: Mendelian randomisation
MR-PRESSO: MR-Pleiotropy RESidual Sum and Outlier
OR: Odds ratio
PE: Pulmonary embolism
RCT: Randomized controlled trial
SNPs: Single nucleotide polymorphisms
TV: Television
VTE: Venous thromboembolism
