# Supplementary material for: Leisure sedentary behaviour increases the risk of venous thromboembolism: a Mendelian randomisation study
Source: BMC Cardiovasc Disord. 2023 Jul 18;23:362. doi: 10.1186/s12872-023-03395-5 (PMC10354998; doi:10.1186/s12872-023-03395-5)
Supplement: Supplementary file 1 — Additional file 1: Supplementary Figure 1. Scatter plot from genetically predicted sedentary TV watching on risk of VTE. Supplementary Figure 2. Scatter plot from genetically predicted sedentary computer use on risk of VTE. Supplementary Figure 3. Scatter plot from genetically predicted sedentary driving on risk of VTE. Supplementary Figure 4. Leave-one-out plot from genetically predicted sedentary TV watching on risk of VTE. Supplementary Figure 5. Leave-one-out plot from genetically predicted sedentary computer use on risk of VTE. Supplementary Figure 6. Leave-one-out plot from genetically predicted sedentary driving on risk of VTE. Supplementary Figure 7. Funnel plot from genetically predicted sedentary TV watching on risk of VTE. Supplementary Figure 8. Funnel plot from genetically predicted sedentary computer use on risk of VTE. Supplementary Figure 9. Funnel plot from genetically predicted sedentary driving on risk of VTE. Supplementary Figure 10. Forest plot from genetically predicted sedentary TV watching on risk of VTE. Supplementary Figure 11. Forest plot from genetically predicted sedentary computer use on risk of VTE. Supplementary Figure 12. Forest plot from genetically predicted sedentary driving on risk of VTE. Supplementary Figure 13. Forest plot of the meta-analysis for genetically predicted sedentary TV watching on risk of VTE in FinnGen and UK Biobank populations. Supplementary Table 1. Summary information on the excluded SNPs for LSB in the present MR study. Supplementary Table 2. Summary information on the SNPs used as genetic instruments for LSB in the present MR study. Supplementary Table 3. MR-PRESSO results. Supplementary Table 4. F-statistics of LSB and physical activity. Supplementary Table 5. MR estimates of the causal relationship between black hair color, physical activity and the risk of VTE. Supplementary Table 6. MRSamePopTest in the FinnGen and UK Biobank population. [file 12872_2023_3395_MOESM1_ESM.docx]

Supplementary Information

**Leisure Sedentary Behaviour Increases the Risk of Venous Thromboembolism: A Mendelian Randomisation Study**

**Table of contents**

[Supplementary Figures 4](#_Toc127217025)

[Supplementary Figure 1. Scatter plot from genetically predicted sedentary TV watching on risk of VTE. 4](#_Toc127217026)

[Supplementary Figure 2. Scatter plot from genetically predicted sedentary computer use on risk of VTE. 5](#_Toc127217027)

[Supplementary Figure 3. Scatter plot from genetically predicted sedentary driving on risk of VTE. 6](#_Toc127217028)

[Supplementary Figure 4. Leave-one-out plot from genetically predicted sedentary TV watching on risk of VTE. 7](#_Toc127217029)

[Supplementary Figure 5. Leave-one-out plot from genetically predicted sedentary computer use on risk of VTE. 8](#_Toc127217030)

[Supplementary Figure 6. Leave-one-out plot from genetically predicted sedentary driving on risk of VTE. 9](#_Toc127217031)

[Supplementary Figure 7. Funnel plot from genetically predicted sedentary TV watching on risk of VTE. 10](#_Toc127217032)

[Supplementary Figure 8. Funnel plot from genetically predicted sedentary computer use on risk of VTE. 11](#_Toc127217033)

[Supplementary Figure 9. Funnel plot from genetically predicted sedentary driving on risk of VTE. 12](#_Toc127217034)

[Supplementary Figure 10. Forest plot from genetically predicted sedentary TV watching on risk of VTE. 13](#_Toc127217035)

[Supplementary Figure 11. Forest plot from genetically predicted sedentary computer use on risk of VTE. 14](#_Toc127217036)

[Supplementary Figure 12. Forest plot from genetically predicted sedentary driving on risk of VTE. 15](#_Toc127217037)

[Supplementary Figure 13. Forest plot of the meta-analysis for genetically predicted sedentary TV watching on risk of VTE in FinnGen and UK Biobank populations. 16](#_Toc127217038)

[Supplementary Tables 17](#_Toc127217039)

[Supplementary Table 1. Summary information on the excluded SNPs for LSB in the present MR study. 17](#_Toc127217040)

[Supplementary Table 2. Summary information on the SNPs used as genetic instruments for LSB in the present MR study. 19](#_Toc127217041)

[Supplementary Table 3. MR-PRESSO results. 27](#_Toc127217042)

[Supplementary Table 4. F-statistics of LSB and physical activity. 27](#_Toc127217043)

[Supplementary Table 5. MR estimates of the causal relationship between black hair color, physical activity and the risk of VTE. 28](#_Toc127217044)

[Supplementary Table 6. MRSamePopTest in the FinnGen and UK Biobank population. 29](#_Toc127217045)

# Supplementary Figures

**
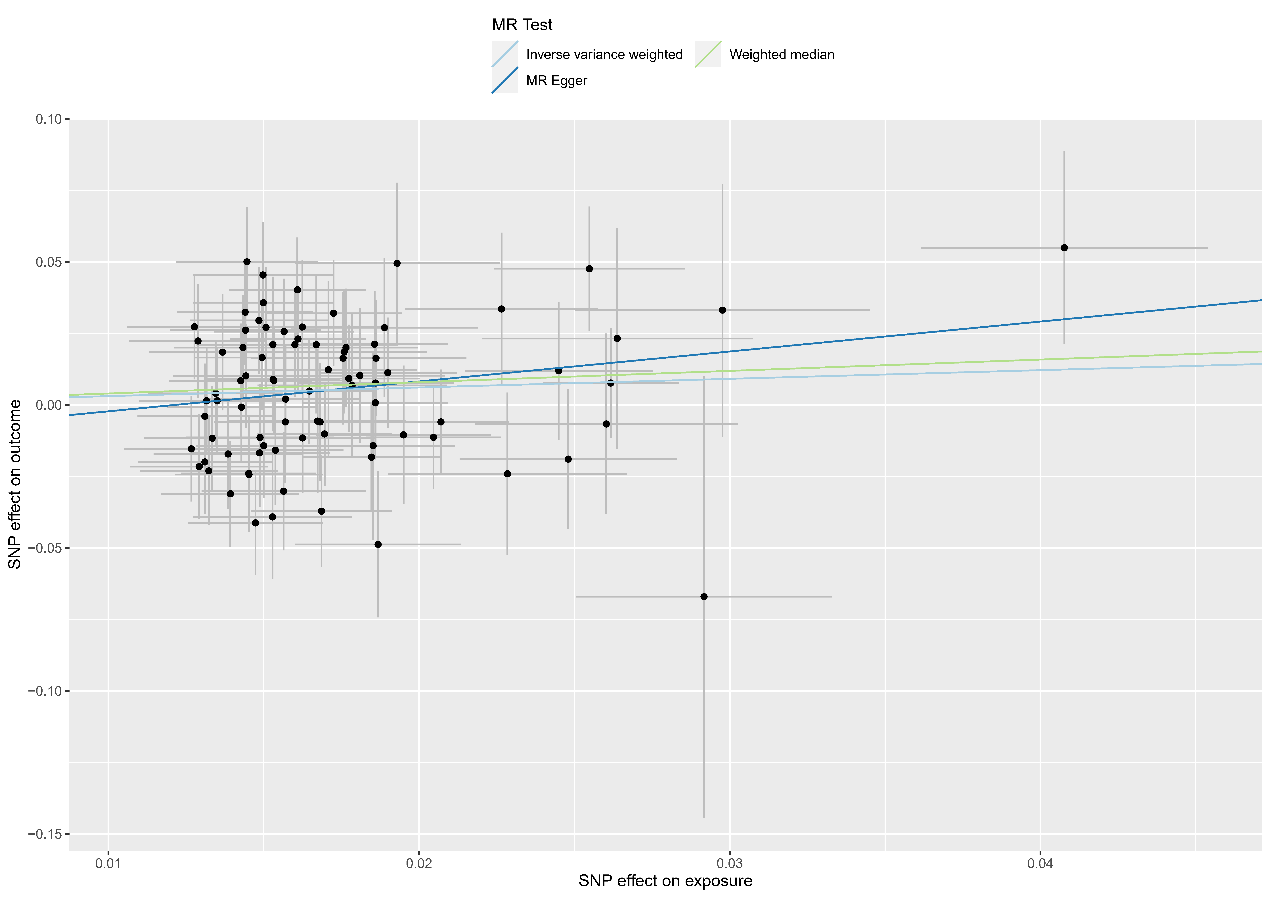
**

## Supplementary Figure 1. Scatter plot from genetically predicted sedentary TV watching on risk of VTE.


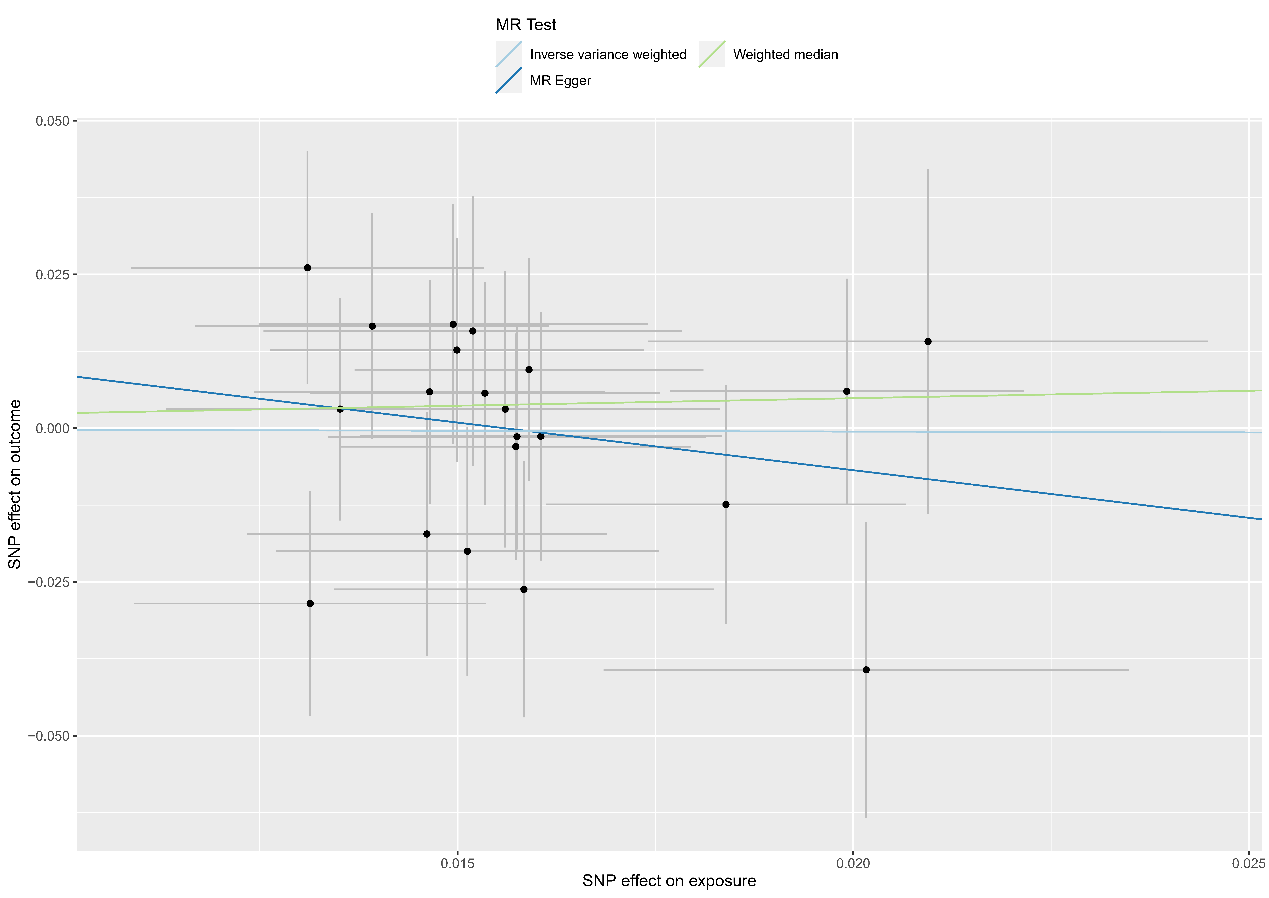


## Supplementary Figure 2. Scatter plot from genetically predicted sedentary computer use on risk of VTE.

**
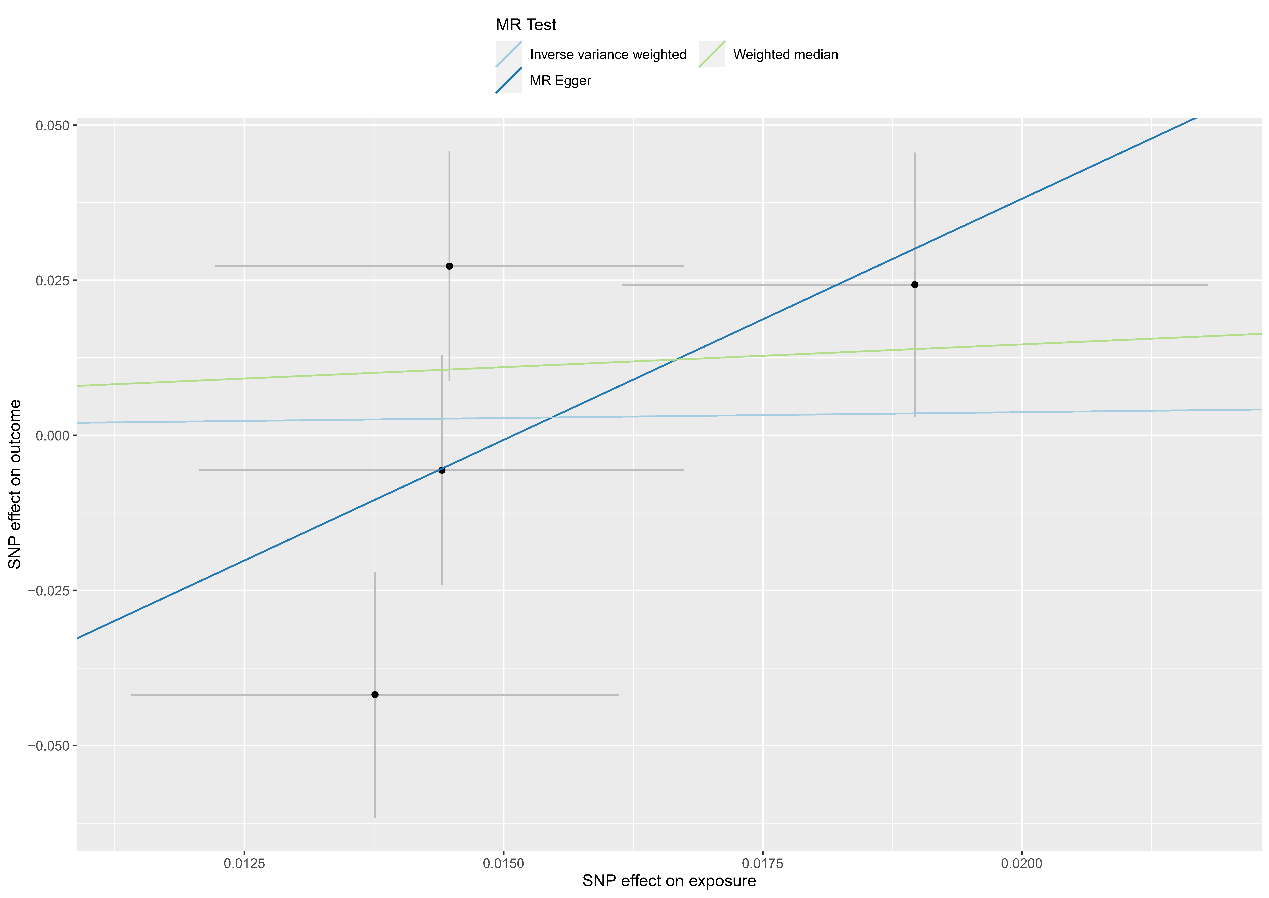
**

## Supplementary Figure 3. Scatter plot from genetically predicted sedentary driving on risk of VTE.

**
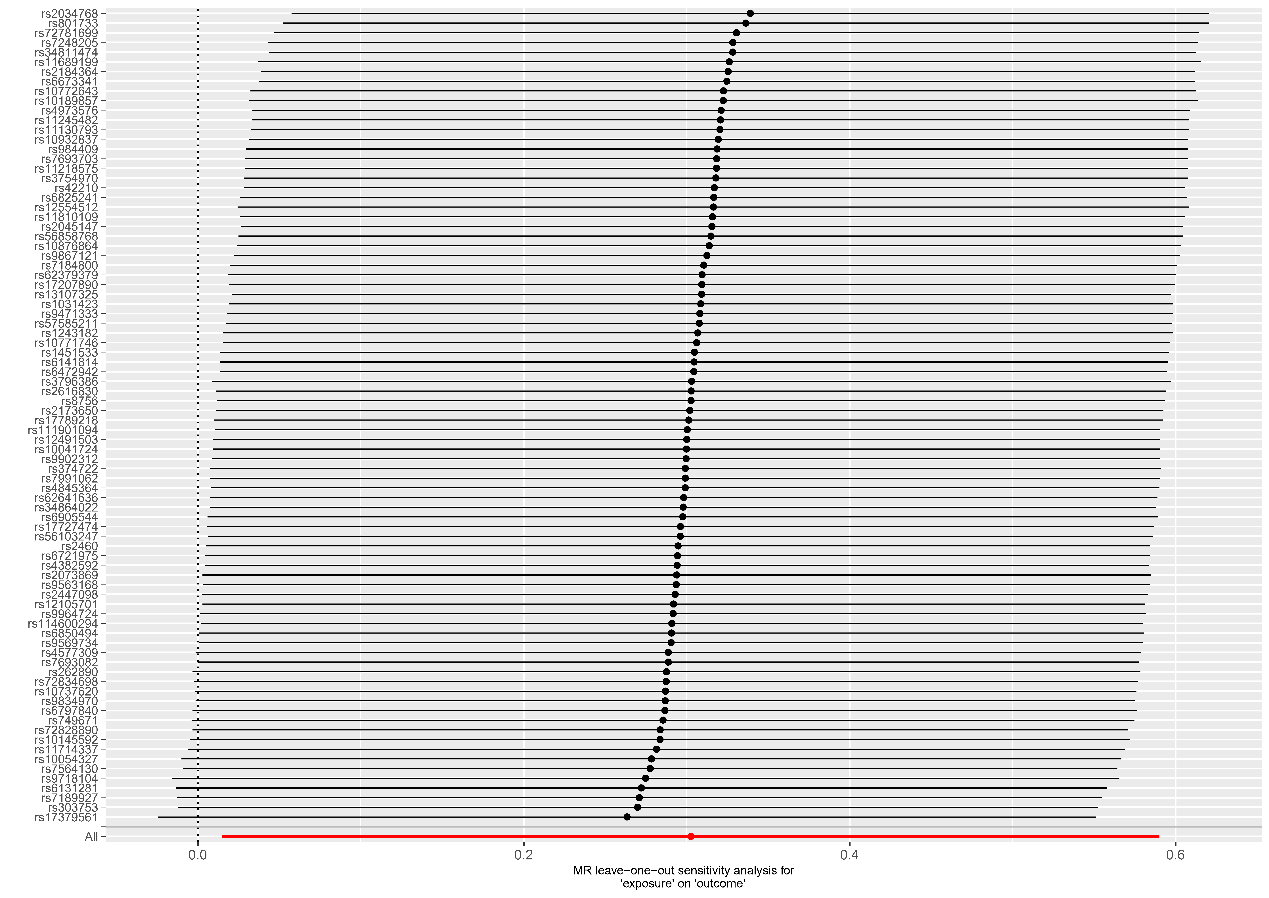
**

## Supplementary Figure 4. Leave-one-out plot from genetically predicted sedentary TV watching on risk of VTE.


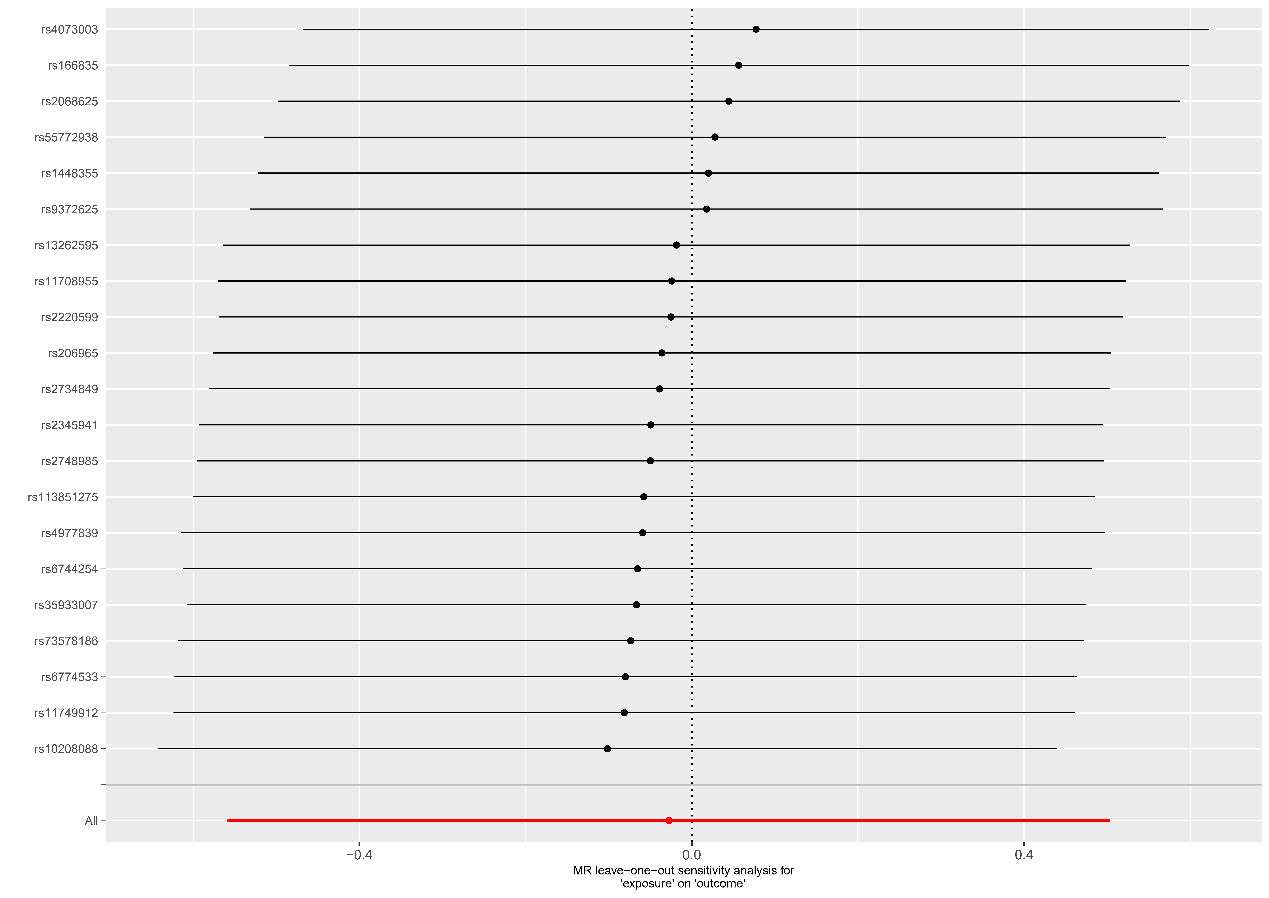


## Supplementary Figure 5. Leave-one-out plot from genetically predicted sedentary computer use on risk of VTE.


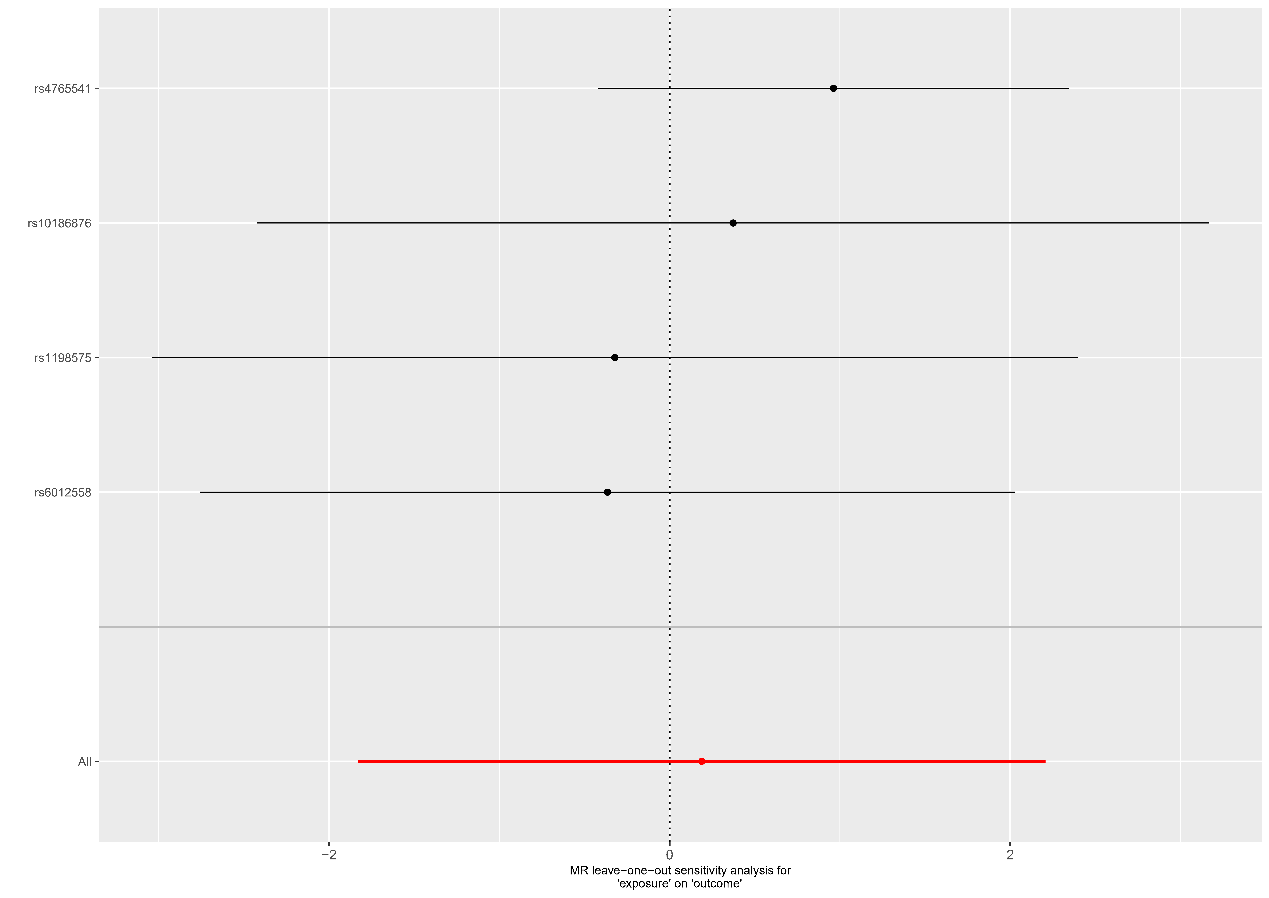


## Supplementary Figure 6. Leave-one-out plot from genetically predicted sedentary driving on risk of VTE.

**
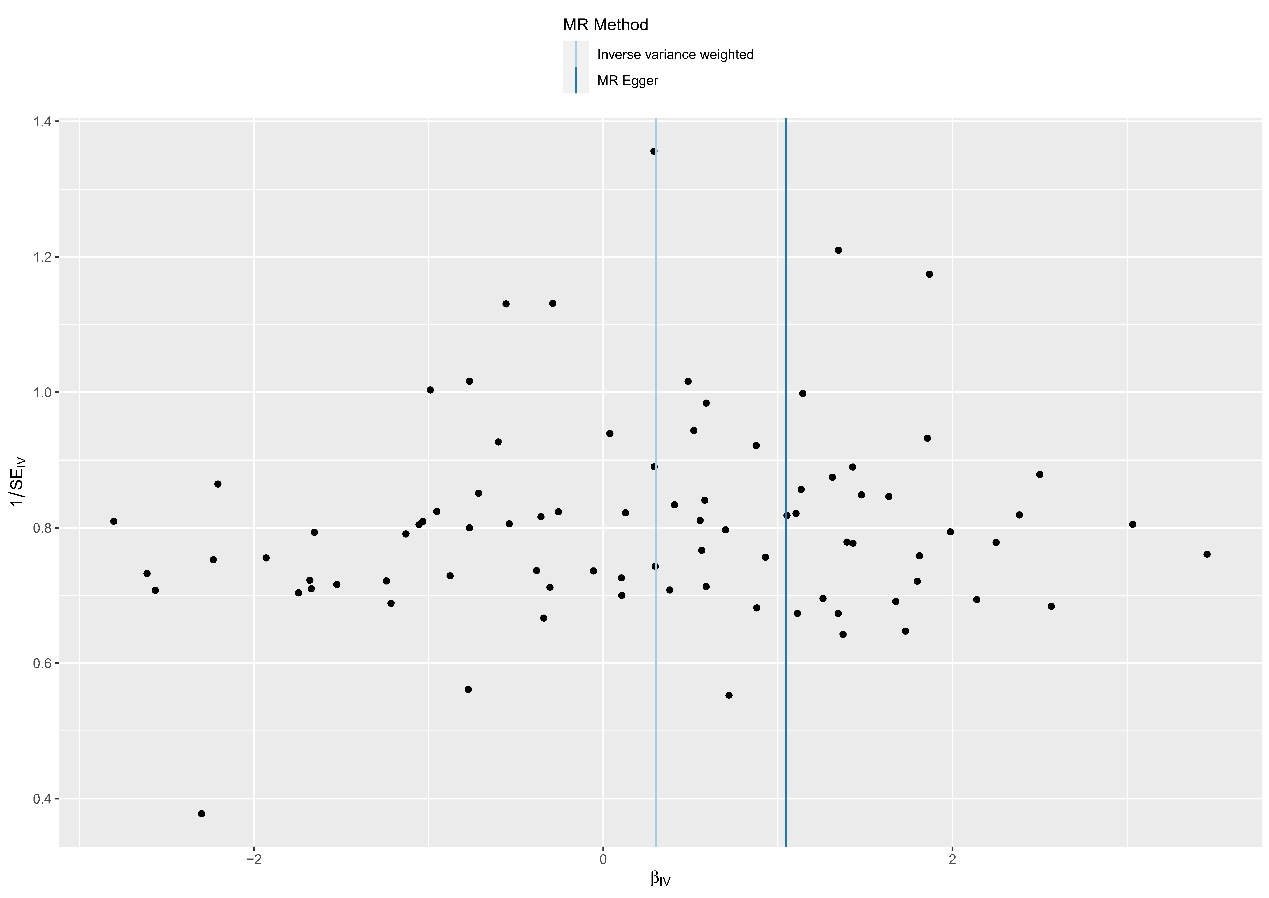
**

## Supplementary Figure 7. Funnel plot from genetically predicted sedentary TV watching on risk of VTE.

**
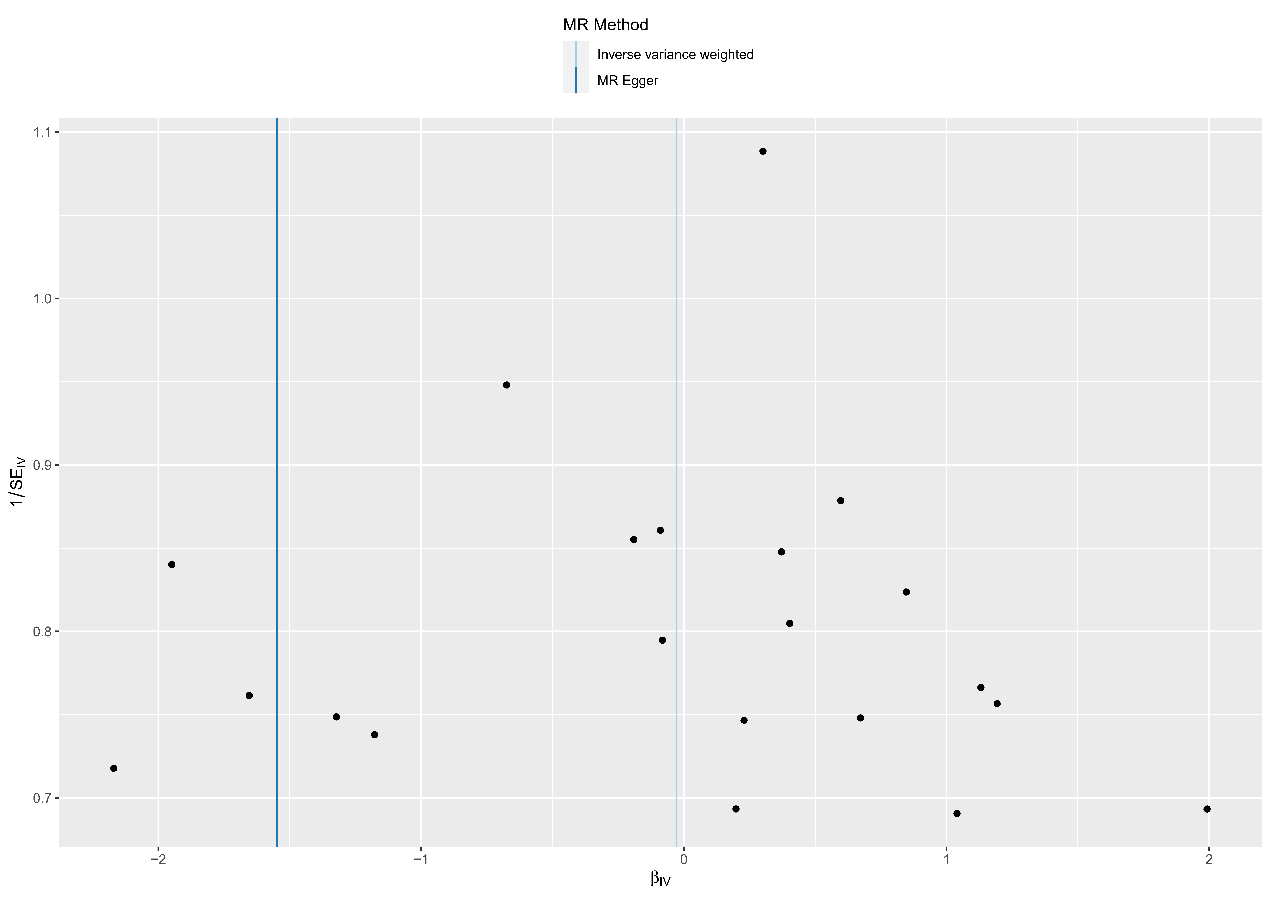
**

## Supplementary Figure 8. Funnel plot from genetically predicted sedentary computer use on risk of VTE.

**
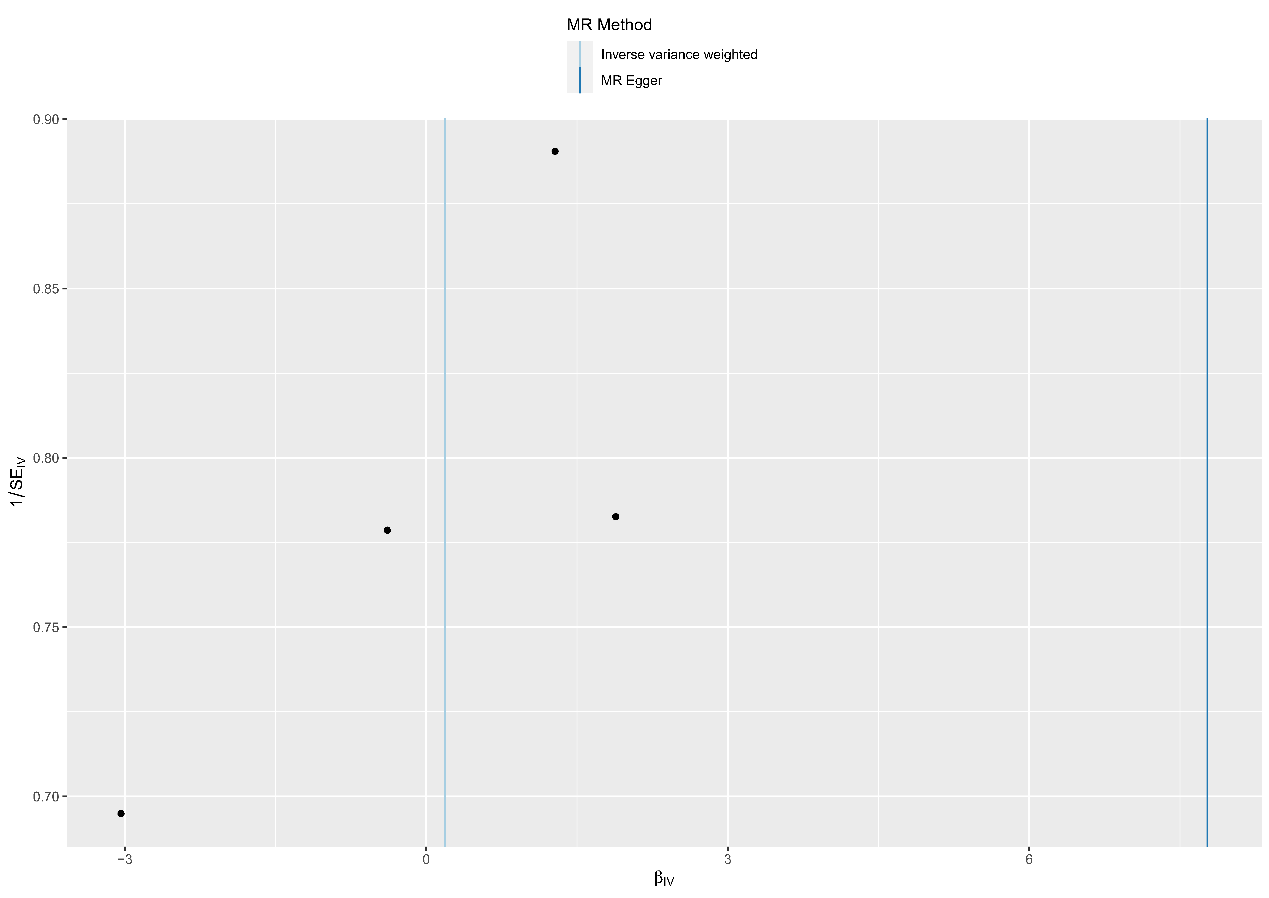
**

## Supplementary Figure 9. Funnel plot from genetically predicted sedentary driving on risk of VTE.

**
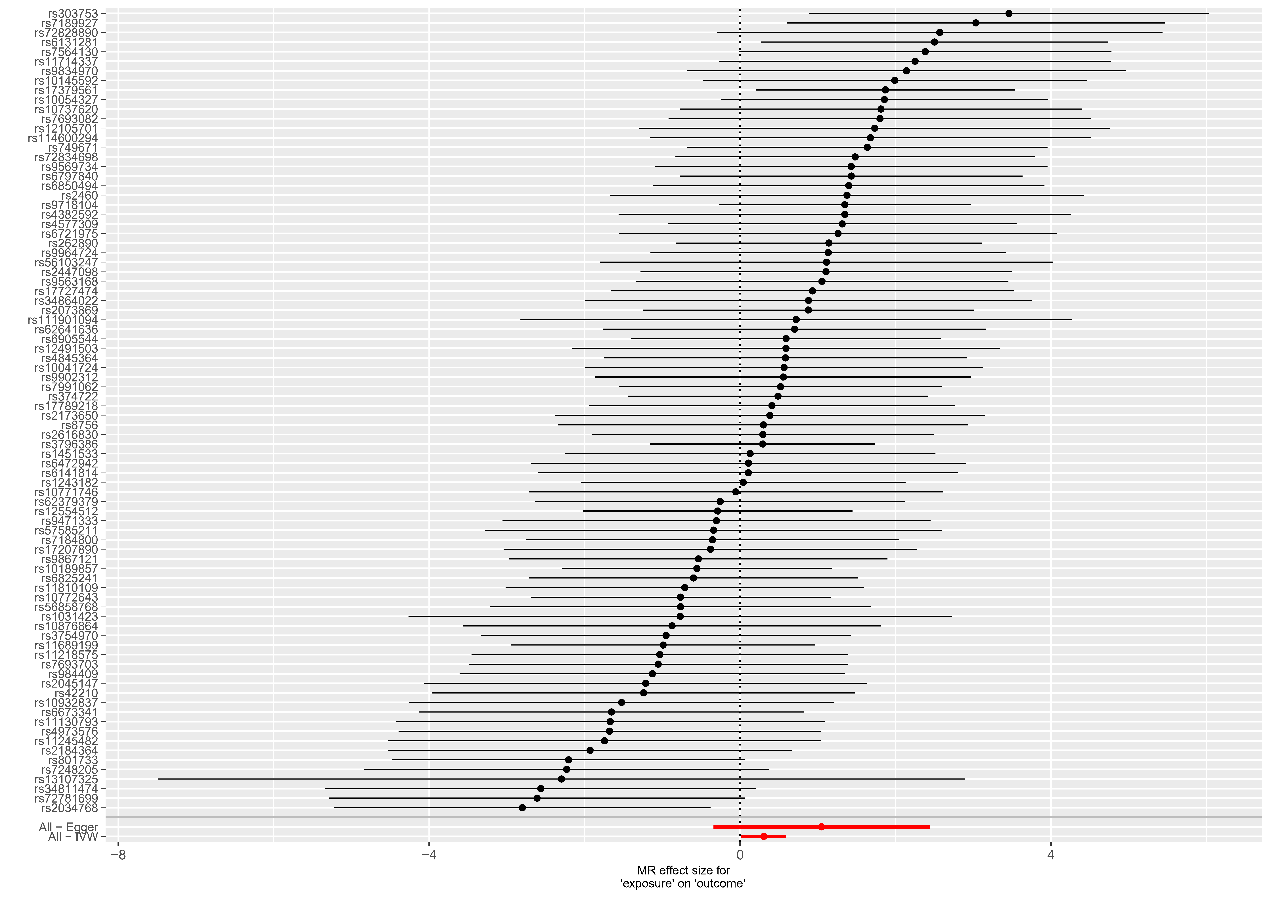
**

## Supplementary Figure 10. Forest plot from genetically predicted sedentary TV watching on risk of VTE.

**
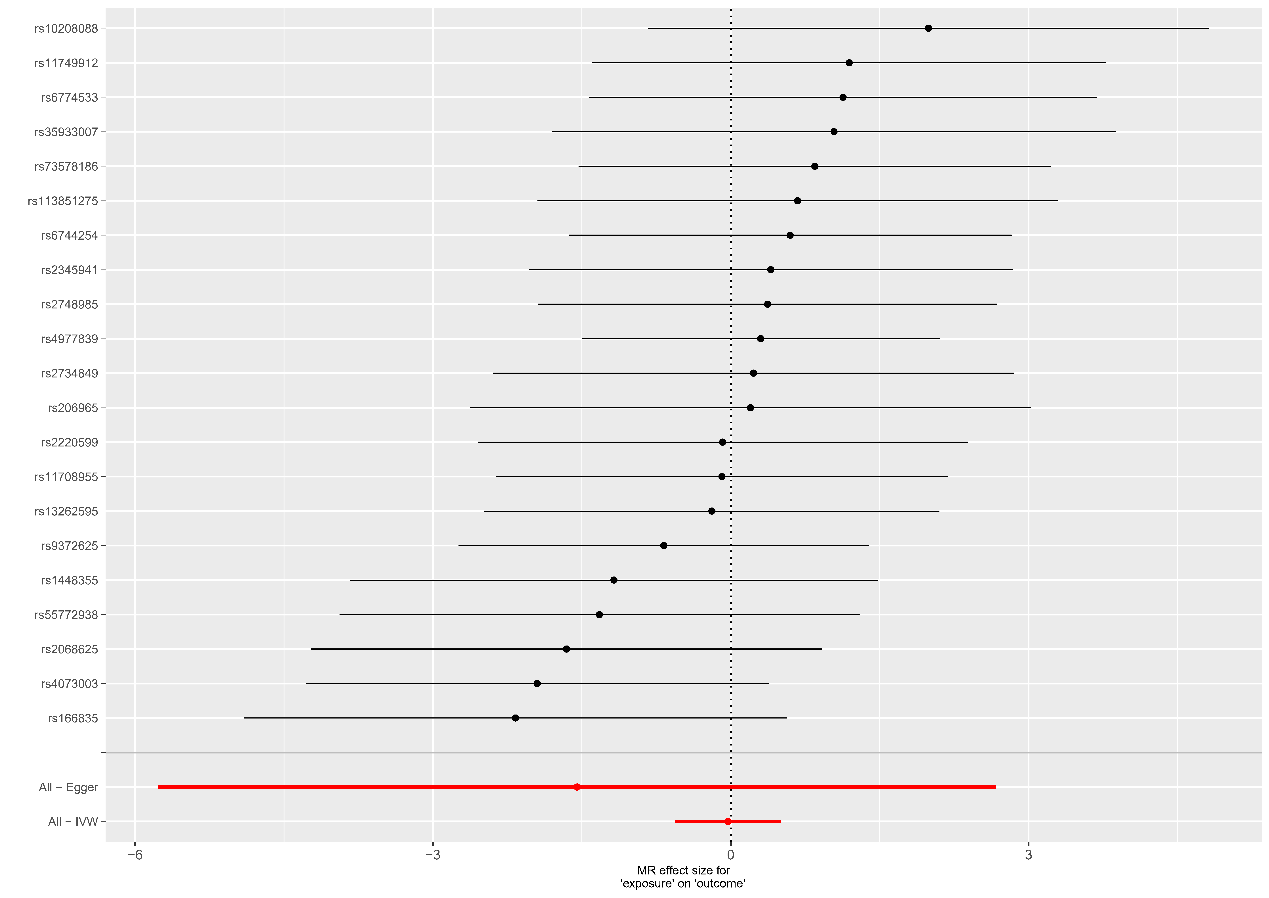
**

## Supplementary Figure 11. Forest plot from genetically predicted sedentary computer use on risk of VTE.

**
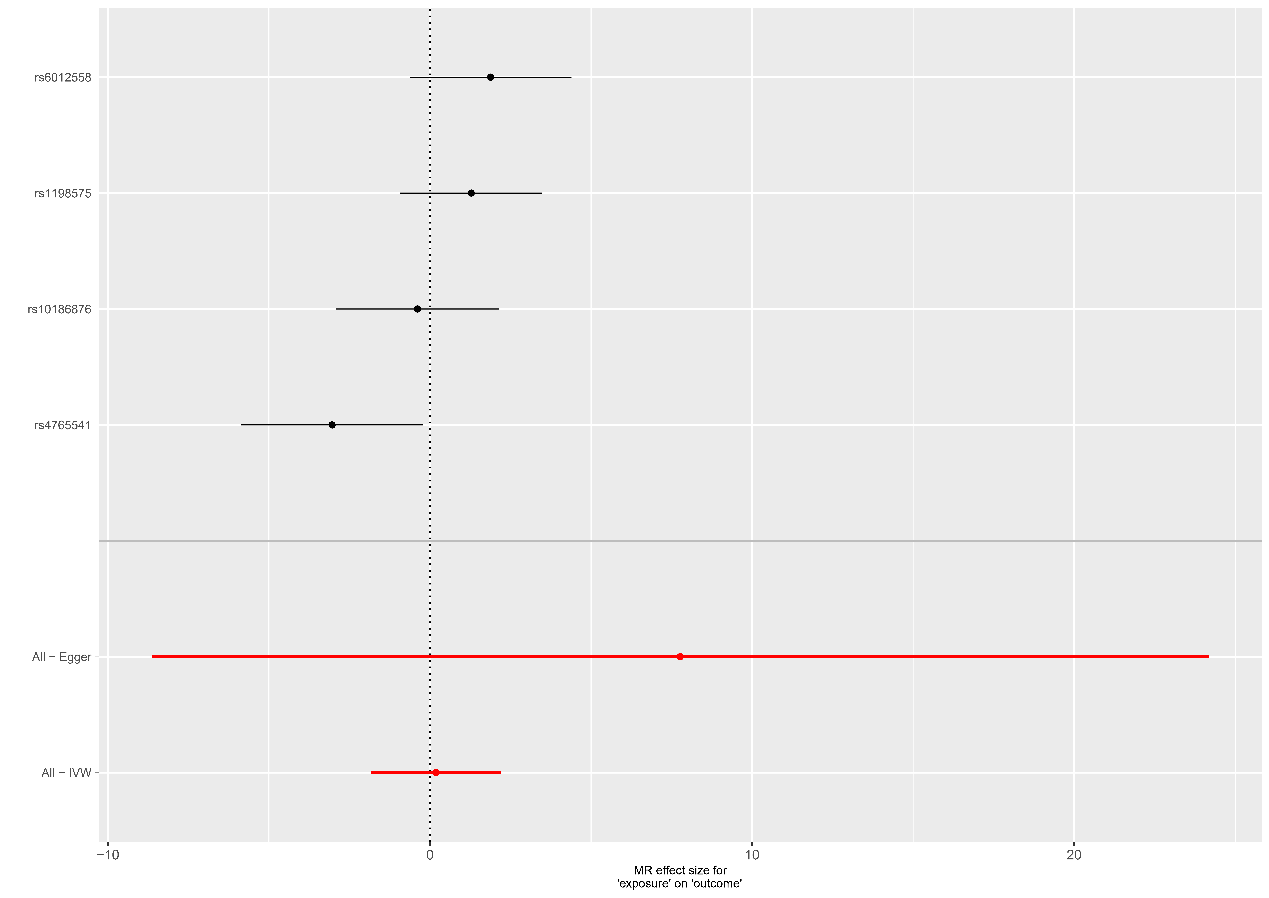
**

## Supplementary Figure 12. Forest plot from genetically predicted sedentary driving on risk of VTE.

**
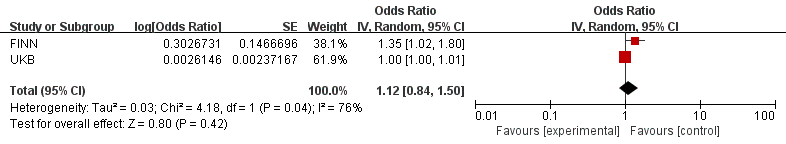
**

## Supplementary Figure 13. Forest plot of the meta-analysis for genetically predicted sedentary TV watching on risk of VTE in FinnGen and UK Biobank populations.

# Supplementary Tables

## Supplementary Table 1. Summary information on the excluded SNPs for LSB in the present MR study.

| Exposure | Number of excluded SNPs | SNP | Effect allele | Other allele | EAF | beta | se | *P* value | Reasons for exclusion |
| --- | --- | --- | --- | --- | --- | --- | --- | --- | --- |
| TV watching | 11 | rs142710267 | T | G | 0.65 | 0.016 | 0.002 | 6.60E-12 | Missing required information for MR tests |
|  |  | rs2584597 | T | C | 0.66 | 0.015 | 0.002 | 2.90E-10 | Missing required information for MR tests |
|  |  | rs66852340 | C | T | 0.78 | -0.018 | 0.003 | 7.90E-12 | Incompatible alleles |
|  |  | rs7716447 | A | G | 0.64 | -0.013 | 0.002 | 3.60E-09 | Incompatible alleles |
|  |  | rs7834121 | G | T | 0.5 | -0.014 | 0.002 | 1.10E-10 | Incompatible alleles |
|  |  | rs10786658 | A | T | 0.41 | -0.014 | 0.002 | 4.60E-11 | Palindromic with MAF close to 0.5 |
|  |  | rs17568389 | T | A | 0.49 | 0.015 | 0.002 | 8.60E-13 | Palindromic with MAF close to 0.5 |
|  |  | rs61331678 | G | C | 0.57 | 0.015 | 0.002 | 6.10E-12 | Palindromic with MAF close to 0.5 |
|  |  | rs62471080 | G | C | 0.54 | -0.013 | 0.002 | 3.70E-09 | Palindromic with MAF close to 0.5 |
|  |  | rs7043521 | A | T | 0.43 | 0.014 | 0.002 | 6.50E-11 | Palindromic with MAF close to 0.5 |
|  |  | rs870151 | T | A | 0.53 | -0.016 | 0.002 | 8.80E-13 | Palindromic with MAF close to 0.5 |
| Computer use | 2 | rs631130 | A | G | 0.44 | 0.013 | 0.002 | 4.00E-09 | Incompatible alleles |
|  |  | rs984409 | A | T | 0.36 | -0.014 | 0.002 | 9.30E-10 | Incompatible alleles |
| Driving | 0 | NA | NA | NA | NA | NA | NA | NA | NA |

MR, Mendelian randomisation; LSB, leisure sedentary behaviours; SNP, single nucleotide polymorphism; TV, television; MAF, minor allele frequency; se, standard error of beta coefficient; EAF, effect allele frequency.

## Supplementary Table 2. Summary information on the SNPs used as genetic instruments for LSB in the present MR study.

| Exposure | Number of SNPs for IVs | SNP | Effect allele | Other allele | EAF | beta | se | *P* value |
| --- | --- | --- | --- | --- | --- | --- | --- | --- |
| TV watching | 84 | rs11810109 | A | T | 0.7 | 0.016 | 0.002 | 5.40E-12 |
|  |  | rs984409 | G | A | 0.36 | -0.015 | 0.002 | 4.10E-11 |
|  |  | rs17379561 | A | T | 0.86 | -0.025 | 0.003 | 1.10E-16 |
|  |  | rs4845364 | A | G | 0.5 | -0.015 | 0.002 | 1.30E-12 |
|  |  | rs6673341 | T | G | 0.47 | -0.015 | 0.002 | 2.20E-11 |
|  |  | rs10737620 | T | A | 0.27 | 0.014 | 0.002 | 2.60E-09 |
|  |  | rs6721975 | T | C | 0.23 | -0.017 | 0.003 | 1.70E-10 |
|  |  | rs72781699 | G | A | 0.8 | -0.019 | 0.003 | 3.00E-12 |
|  |  | rs12105701 | C | T | 0.4 | -0.013 | 0.002 | 5.40E-09 |
|  |  | rs7564130 | T | C | 0.64 | -0.015 | 0.002 | 2.80E-11 |
|  |  | rs10189857 | A | G | 0.57 | -0.02 | 0.002 | 6.20E-21 |
|  |  | rs62641636 (replaced by rs62145959) | A | G | 0.69 | 0.014 | 0.002 | 7.00E-10 |
|  |  | rs11689199 | A | G | 0.6 | 0.018 | 0.002 | 5.50E-17 |
|  |  | rs1451533 | G | A | 0.72 | -0.016 | 0.002 | 1.10E-10 |
|  |  | rs374722 | G | A | 0.15 | 0.024 | 0.003 | 5.50E-16 |
|  |  | rs3754970 | T | C | 0.5 | -0.015 | 0.002 | 4.80E-12 |
|  |  | rs4577309 | A | G | 0.47 | 0.016 | 0.002 | 1.60E-13 |
|  |  | rs10932837 | C | T | 0.49 | -0.013 | 0.002 | 1.30E-09 |
|  |  | rs4973576 | C | A | 0.3 | -0.015 | 0.002 | 1.00E-09 |
|  |  | rs9834970 | T | C | 0.5 | 0.013 | 0.002 | 3.30E-09 |
|  |  | rs3796386 | G | A | 0.57 | -0.026 | 0.002 | 3.20E-33 |
|  |  | rs11130793 | C | T | 0.6 | 0.013 | 0.002 | 5.00E-09 |
|  |  | rs11714337 | G | A | 0.57 | 0.014 | 0.002 | 4.70E-11 |
|  |  | rs6797840 | A | C | 0.46 | -0.016 | 0.002 | 1.70E-13 |
|  |  | rs2034768 | A | G | 0.49 | 0.015 | 0.002 | 8.60E-12 |
|  |  | rs9867121 | C | A | 0.82 | 0.019 | 0.003 | 3.90E-12 |
|  |  | rs12491503 | G | A | 0.67 | -0.014 | 0.002 | 5.50E-10 |
|  |  | rs114600294 | G | C | 0.79 | -0.016 | 0.003 | 7.90E-10 |
|  |  | rs34811474 | G | A | 0.77 | 0.015 | 0.003 | 2.30E-09 |
|  |  | rs6850494 | A | C | 0.62 | -0.014 | 0.002 | 1.10E-10 |
|  |  | rs13107325 | C | T | 0.93 | -0.029 | 0.004 | 1.50E-12 |
|  |  | rs7693703 | G | A | 0.91 | 0.023 | 0.004 | 2.70E-09 |
|  |  | rs6825241 | C | A | 0.54 | -0.017 | 0.002 | 4.90E-15 |
|  |  | rs7693082 | G | C | 0.3 | 0.015 | 0.002 | 1.70E-10 |
|  |  | rs262890 | A | G | 0.7 | -0.019 | 0.002 | 3.20E-15 |
|  |  | rs1031423 | T | C | 0.22 | -0.019 | 0.003 | 1.80E-12 |
|  |  | rs57585211 | T | G | 0.83 | -0.017 | 0.003 | 4.30E-09 |
|  |  | rs10041724 | T | C | 0.81 | 0.018 | 0.003 | 3.90E-11 |
|  |  | rs62379379 | G | T | 0.93 | -0.026 | 0.004 | 7.80E-10 |
|  |  | rs10054327 | G | A | 0.58 | 0.017 | 0.002 | 3.40E-15 |
|  |  | rs42210 | G | C | 0.29 | -0.014 | 0.002 | 7.30E-09 |
|  |  | rs72828890 | C | T | 0.87 | 0.019 | 0.003 | 4.90E-09 |
|  |  | rs72834698 | G | A | 0.86 | 0.023 | 0.003 | 2.70E-13 |
|  |  | rs9471333 | C | T | 0.45 | 0.013 | 0.002 | 1.50E-09 |
|  |  | rs6905544 | A | G | 0.4 | -0.019 | 0.002 | 8.50E-18 |
|  |  | rs17789218 | T | C | 0.76 | 0.019 | 0.003 | 1.40E-13 |
|  |  | rs2184364 (replaced by rs12214573) | A | G | 0.78 | 0.016 | 0.003 | 3.00E-09 |
|  |  | rs9718104 | T | G | 0.94 | -0.041 | 0.005 | 9.30E-19 |
|  |  | rs6472942 | T | C | 0.57 | -0.013 | 0.002 | 1.80E-09 |
|  |  | rs2616830 | G | A | 0.46 | 0.016 | 0.002 | 2.90E-14 |
|  |  | rs34864022 | A | G | 0.93 | -0.026 | 0.004 | 1.40E-09 |
|  |  | rs12554512 | T | C | 0.58 | 0.021 | 0.002 | 3.80E-21 |
|  |  | rs4382592 | T | G | 0.3 | 0.014 | 0.002 | 6.70E-09 |
|  |  | rs2073869 | C | T | 0.83 | 0.019 | 0.003 | 1.50E-10 |
|  |  | rs1243182 | C | T | 0.69 | -0.019 | 0.002 | 2.00E-15 |
|  |  | rs2045147 | A | G | 0.45 | 0.013 | 0.002 | 5.90E-09 |
|  |  | rs11245482 | T | C | 0.61 | -0.013 | 0.002 | 2.60E-09 |
|  |  | rs17727474 | C | T | 0.83 | 0.018 | 0.003 | 3.10E-09 |
|  |  | rs801733 | A | C | 0.64 | 0.017 | 0.002 | 7.30E-14 |
|  |  | rs17207890 | G | A | 0.66 | 0.016 | 0.002 | 6.70E-12 |
|  |  | rs11218575 | C | T | 0.57 | 0.015 | 0.002 | 2.10E-12 |
|  |  | rs10772643 | C | T | 0.11 | 0.025 | 0.003 | 1.30E-12 |
|  |  | rs10771746 | C | T | 0.72 | -0.014 | 0.002 | 2.50E-09 |
|  |  | rs10876864 | G | A | 0.43 | -0.013 | 0.002 | 1.00E-09 |
|  |  | rs8756 | C | A | 0.48 | -0.013 | 0.002 | 5.30E-10 |
|  |  | rs2173650 | G | T | 0.85 | 0.018 | 0.003 | 4.60E-09 |
|  |  | rs9563168 | G | A | 0.79 | 0.018 | 0.003 | 4.30E-11 |
|  |  | rs9569734 (replaced by rs9569733) | A | G | 0.84 | 0.019 | 0.003 | 3.40E-10 |
|  |  | rs56858768 | G | A | 0.7 | -0.015 | 0.002 | 3.40E-10 |
|  |  | rs7991062 | C | G | 0.66 | -0.018 | 0.002 | 8.80E-15 |
|  |  | rs10145592 | C | G | 0.41 | -0.015 | 0.002 | 1.80E-11 |
|  |  | rs2460 | G | A | 0.74 | -0.015 | 0.002 | 5.00E-10 |
|  |  | rs7189927 | T | C | 0.36 | 0.015 | 0.002 | 3.40E-11 |
|  |  | rs749671 | G | A | 0.63 | 0.016 | 0.002 | 2.70E-12 |
|  |  | rs7184800 | G | A | 0.7 | 0.017 | 0.002 | 8.20E-13 |
|  |  | rs2447098 | C | A | 0.48 | -0.015 | 0.002 | 6.80E-12 |
|  |  | rs9902312 | T | C | 0.68 | 0.015 | 0.002 | 4.50E-11 |
|  |  | rs303753 | G | A | 0.65 | -0.014 | 0.002 | 2.70E-10 |
|  |  | rs9964724 | C | T | 0.32 | 0.018 | 0.002 | 3.30E-14 |
|  |  | rs7248205 | C | T | 0.4 | 0.014 | 0.002 | 3.40E-10 |
|  |  | rs111901094 (replaced by rs79954596) | G | T | 0.82 | -0.017 | 0.003 | 2.00E-09 |
|  |  | rs6131281 | C | T | 0.6 | 0.016 | 0.002 | 3.10E-13 |
|  |  | rs6141814 | C | A | 0.61 | -0.014 | 0.002 | 1.30E-09 |
|  |  | rs56103247 | C | T | 0.94 | 0.03 | 0.005 | 3.80E-10 |
| Computer use | 21 | rs2748985 | T | C | 0.45 | -0.015 | 0.002 | 4.10E-12 |
|  |  | rs6744254 | C | T | 0.47 | -0.016 | 0.002 | 5.40E-13 |
|  |  | rs10208088 | C | T | 0.42 | 0.013 | 0.002 | 4.40E-09 |
|  |  | rs11708955 | T | C | 0.69 | -0.016 | 0.002 | 4.20E-11 |
|  |  | rs6774533 | C | T | 0.29 | -0.015 | 0.002 | 1.20E-09 |
|  |  | rs2068625 | T | C | 0.3 | -0.016 | 0.002 | 4.00E-11 |
|  |  | rs2220599 | C | G | 0.63 | -0.016 | 0.002 | 2.40E-12 |
|  |  | rs11749912 | A | G | 0.42 | 0.014 | 0.002 | 4.90E-10 |
|  |  | rs9372625 | G | A | 0.62 | -0.018 | 0.002 | 5.70E-16 |
|  |  | rs55772938 | A | G | 0.7 | -0.015 | 0.002 | 3.90E-10 |
|  |  | rs2345941 | A | G | 0.55 | 0.015 | 0.002 | 4.00E-11 |
|  |  | rs13262595 | A | G | 0.44 | -0.016 | 0.002 | 1.20E-12 |
|  |  | rs4977839 | G | A | 0.58 | -0.02 | 0.002 | 4.70E-19 |
|  |  | rs113851275 | G | A | 0.89 | -0.021 | 0.004 | 3.20E-09 |
|  |  | rs73578186 | C | T | 0.68 | 0.015 | 0.002 | 2.30E-10 |
|  |  | rs2734849 | A | G | 0.49 | -0.014 | 0.002 | 8.20E-10 |
|  |  | rs1448355 | C | T | 0.38 | -0.015 | 0.002 | 1.30E-10 |
|  |  | rs35933007 | G | A | 0.77 | -0.015 | 0.003 | 9.20E-09 |
|  |  | rs206965 | T | C | 0.21 | 0.016 | 0.003 | 9.10E-09 |
|  |  | rs166835 | C | T | 0.44 | 0.013 | 0.002 | 3.40E-09 |
|  |  | rs4073003 | A | G | 0.87 | 0.02 | 0.003 | 1.20E-09 |
| Driving | 4 | rs1198575 | T | C | 0.19 | 0.019 | 0.003 | 2.00E-11 |
|  |  | rs10186876 | A | G | 0.36 | 0.014 | 0.002 | 7.20E-10 |
|  |  | rs4765541 | T | C | 0.66 | 0.014 | 0.002 | 5.10E-09 |
|  |  | rs6012558 | G | A | 0.58 | 0.014 | 0.002 | 1.60E-10 |

MR, Mendelian randomisation; LSB, leisure sedentary behaviours; SNP, single nucleotide polymorphism; IVs, instrumental variables; TV, television; se, standard error of beta coefficient; EAF, effect allele frequency.

## Supplementary Table 3. MR-PRESSO results.

| Exposure | Observation value | *P* value |
| --- | --- | --- |
| TV watching | 111.347 | 0.110 |
| Computer use | 16.465 | 0.860 |
| Driving | 13.217 | 0.061 |

MR-PRESSO, MR-Pleiotropy RESidual Sum and Outlier

## Supplementary Table 4. F-statistics of LSB and physical activity.

| Exposure | Number of SNPs | *F* |
| --- | --- | --- |
| TV watching | 84 | 48 |
| Computer use | 21 | 44 |
| Driving | 4 | 47 |
| Physical activity | 10 | 41 |

## Supplementary Table 5. MR estimates of the causal relationship between black hair color, physical activity and the risk of VTE.

| Exposure | Number of SNPs | Methods | *β* | *se* | *p*-value |
| --- | --- | --- | --- | --- | --- |
| Black hair color | 90 | IVW | 0.0020 | 0.0044 | 0.658 |
|  |  | WM | -0.0004 | 0.0052 | 0.938 |
|  |  | MR-Egger | -0.0094 | 0.0224 | 0.674 |
|  |  | Weighted mode | -0.0021 | 0.0117 | 0.856 |
|  |  | Simple mode | -0.0021 | 0.0144 | 0.883 |
| Physical activity | 10 | IVW | -0.0427 | 0.2694 | 0.874 |
|  |  | WM | -0.1119 | 0.3588 | 0.755 |
|  |  | MR-Egger | 0.3329 | 0.5337 | 0.550 |
|  |  | Weighted mode | -0.4245 | 0.6047 | 0.500 |
|  |  | Simple mode | -0.4080 | 0.6307 | 0.534 |

## Supplementary Table 6. MRSamePopTest in the FinnGen and UK Biobank population.

| Exposure | Mean difference | 95% CI | *z* value | *p*-value |
| --- | --- | --- | --- | --- |
| TV watching | 0.0007 | (-0.0041, 0.0056) | 0.30 | 0.110 |
| Computer use | 0.0006 | (-0.0074, 0.0086) | 0.15 | 0.883 |
| Driving | 0.0008 | (-0.0304, 0.0320) | 0.05 | 0.960 |
